# The impact of psychological factors on hypertension and its psychological intervention in pilot selection candidates

**DOI:** 10.3389/fpsyg.2025.1634423

**Published:** 2025-11-06

**Authors:** Xue-ting Dou, Ming-jing Ji, Zhong-yang Sun, Hong-liang Sun, Yuan Wang, Hai-bin Zou, Si-yang Wang, Lin Gong

**Affiliations:** 1 Department of Psychology, Air Force Hospital of Eastern Theater Command, Nanjing, China; 2 Department of Outpatient, Air Force Hospital of Eastern Theater Command, Nanjing, China; 3 Department of Orthopedics, Air Force Hospital of Eastern Theater Command, Nanjing, China; 4 Physical Examination Department of Nanjing Selection Center of Air Force Recruitment, Nanjing, China

**Keywords:** hypertension, anxiety, perceived stress, relaxation training, cognitive behavioral therapy

## Abstract

**Background:**

Individual blood pressure levels can be influenced by psychological factors.

**Objective:**

This study aimed to investigate the relationship between hypertension and psychological states, as well as the underlying mechanisms, using a combination of cross-sectional comparison and experimental intervention.

**Methods:**

A total of 102 individuals with hypertension and 108 individuals with normal blood pressure participating in pilot selection were recruited. Individuals with hypertension were divided into two intervention groups and a control group: one group received relaxation therapy alone, while the other received a combination of relaxation therapy and cognitive training. Questionnaires were administered to assess anxiety levels and perceived stress.

**Results:**

The results showed a significant positive correlation between perceived stress and blood pressure in the BBG, and a marginal positive correlation between anxiety and blood pressure. No such correlations were found in the normal blood pressure group. Pre- and post-intervention analyses indicated that the intervention group exhibited a significant reduction in blood pressure compared to baseline levels, along with a notably higher retest pass rate. However, anxiety levels increased significantly across all groups.

**Conclusion:**

These findings suggest that blood pressure is influenced by anxiety and perceived stress, and psychological interventions can effectively manage blood pressure. However, these interventions may not primarily operate through reducing anxiety, and further research is needed to explore the psychological mechanisms involved.

## 1 Introduction

Hypertension represents a major global health challenge, significantly contributing to cardiovascular disease and mortality (Dzau and Balatbat, 2019). Prolonged exposure to high-stress environments may further elevate the risk of its onset (Hahad et al., 2023). The relationship between occupational characteristics and hypertension has garnered considerable attention, with pilots being recognized as a high-risk group due to their prolonged exposure to high workloads, circadian rhythm disruptions, and high-stress environments (Kang, 2022; Maculewicz et al., 2022). Therefore, blood pressure indicators are very important for the selection of pilots.

Moreover, psychological state influences physiological state. Hypertension as a psychosomatic disorder, with links between perceived stress, anxiety (Mann, 2012; Spruill et al., 2019). A study demonstrated that anxiety modifies sympathetic firing patterns, activating the sympathetic nervous system and the hypothalamic-pituitary-adrenal axis in patients, which results in elevated blood pressure (Lambert et al., 2010; Hamam et al., 2020). These findings have significant clinical implications for the early detection of hypertension and the implementation of psychological interventions (Lim et al., 2021). Psychological interventions, such as relaxation training and cognitive therapy, have been shown to be effective in reducing blood pressure (Dickinson et al., 2008; Li et al., 2021). A recent study involving 64 participants found that relaxation training combined with slow breathing exercises can effectively reduce blood pressure (Pathan et al., 2023). Another study involving 92 participants also found that cognitive-based psychological interventions contribute to reducing blood pressure (Clemow et al., 2018). However, cross-sectional comparisons of different psychological interventions are lacking, and the mechanisms of action are unclear (Tan and Morgan, 2015).

Blood pressure is an important indicator in pilot selection. It can be temporarily elevated by psychological factors such as tension during selection tests. This temporary increase may disqualify candidates from further participation. Therefore, it is necessary to investigate whether reductions in blood pressure following psychological interventions are associated with decreases in anxiety, and to explore effective interventions to minimize transient elevations during the initial screening of pilot selection.

The novelty of this study lies in the recruitment of pilot candidates as the study population. It compared anxiety and stress levels between the Normal Control Group (NCG) and the Hypertension Group (BBG), evaluated the effectiveness of two different psychological interventions in lowering blood pressure, and further explored the underlying psychological mechanisms. This research provides both theoretical and practical support for optimizing medical screening, which is of particular significance for specific occupational groups. The findings may help to reduce the risk of misdiagnosis caused by incomplete assessment of psychological and physiological states, thereby enhancing the accuracy and fairness of medical screening.

## 2 Materials and methods

### 2.1 Study design

**FIGURE 1:**
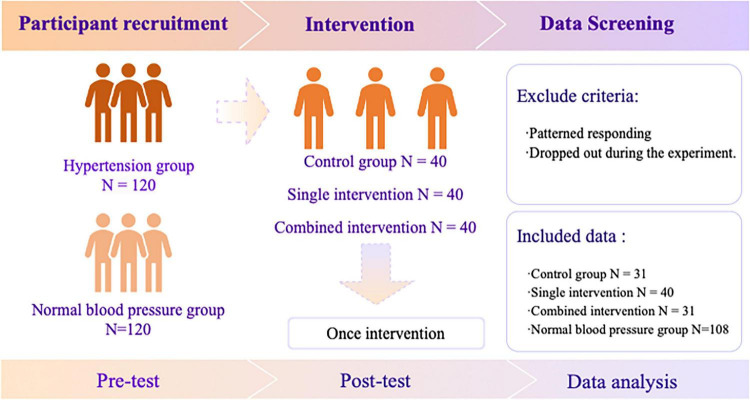
Flowchart of study design.

This study adopted a randomized controlled design to examine the psychological and physiological outcomes of interventions among pilots with hypertension. Participants were allocated into different groups through computer-generated randomization, and the procedures included recruitment, intervention, and follow-up assessments. The overall procedure is illustrated in Figure 1.

### 2.2 Participants

120 male participants with hypertension were recruited from pilot medical examination. Participants were recruited for this study if they met the following criteria: (1) Met the specified range of the 2018 Chinese Hypertension Prevention and Treatment Guidelines, where hypertension is defined as a systolic blood pressure ≥ 140 mm Hg and/or a diastolic blood pressure ≥ 90 mm Hg (1 mm Hg = 0.133 kPa) (Li-Sheng, 2019); (2) had no organic damage such as retinal lesions or electrocardiographic abnormalities; (3) had no history of hypertension or syncope; (4) had no family history of hypertension; (5) had no history of anemia, hyperthyroidism, adrenal cortical adenoma, or other diseases affecting the cardiovascular system.

As a control, 120 healthy male participants were recruited during the pilot medical examination. The studies involving human participants were reviewed and approved by the Medical Ethics Committee of The Air Force Hospital from Eastern Theater of the People’s Liberation Army (PLA).

### 2.3 Instrument

Anxiety was assessed using the Self-Rating Anxiety Scale, a 20-item measure in which items 5, 7, 10, and 13 are reverse-scored (Zung, 1971). A 4-point Likert scale was used, with 1 indicating “never” and 4 signifying “most or all of the time.” Higher scores reflected elevated levels of anxiety in individuals. The Cronbach’s αα in this study was 0.82.

Perceived stress was measured with Chinese Perceived Stress Scale, a 14-item measure in which items 4, 5, 6, 7, 9, 10, 12, and 13 are reverse-scored (Cohen et al., 1983; Yang and Huang, 2003). A 6-point Likert scale was used, with 1 being “never” and 6 being “always.” A higher total score indicates a higher perceived stress and a greater sense of uncontrollability. Cronbach’s α in this study was 0.78.

Blood pressure was measured using the Omron HEM-907XL, with the following standardized procedures: the measurements were taken three times, with a one-min interval between each measurement to ensure accuracy. The appropriate cuff size (22–32 cm) was selected based on the participant’s arm circumference. Participants were instructed to remain seated in a relaxed position, with their feet flat on the floor and their back supported. A resting period of at least five minutes was allowed before measurements were taken to ensure a stable baseline reading.

The pass rate refers to the probability that blood pressure meets the selection criteria for Air Force pilot recruitment.

### 2.4 Intervention

The single intervention group took part in relaxation training sessions lasting about 30 min. These sessions included guided techniques to promote physical and mental relaxation, such as controlled breathing exercises, progressive muscle relaxation and visualization methods to reduce stress and tension.

The combined intervention group received a more comprehensive approach. Participants first underwent a recognized intervention based on cognitive behavioral therapy (CBT). This phase involved structured activities that focused on identifying and challenging negative thought patterns, promoting adaptive coping strategies, and developing emotional regulation skills. This was followed by approximately 30 min of relaxation training, like the single intervention group. This sequential approach aimed to combine the cognitive benefits of CBT with the physiological and emotional relaxation benefits of the training to achieve more substantial and lasting effects on stress and blood pressure reduction. Both interventions were delivered under the guidance of trained professionals in a quiet and comfortable environment to maximize participants’ concentration and effectiveness.

Additionally, the Control group received no intervention.

### 2.5 Data collection

Before the start of the study, all 240 participants signed the informed consent form. Participants in the hypertension group were randomly assigned to their respective groups using the website https://www.randomizer.org/. The entire study was conducted at a dedicated medical examination center. Data were collected by three medical staff members responsible for aviation medical examinations, who were blinded to the study purpose. To ensure consistency, each participant’s psychological and blood pressure data were collected by the same assessor.

During the first blood pressure measurement, participants rested in the waiting room for 5 min prior to measurement, which was usually conducted between 8:00 and 10:00 a.m. In the afternoon, between 2:00 and 3:00 p.m., participants returned according to their assigned groups to receive either a single psychological intervention (approximately 30 min) or a combined psychological intervention (approximately 50 min), while the control group did not receive any intervention. At around 4:30 p.m., all groups rested in the waiting room for 5 min before the second blood pressure measurement.

### 2.6 Data analysis

SPSS 26.0 was used to compare the differences in anxiety, perceived stress, systolic blood pressure (SBP) and diastolic blood pressure (DBP) between the BBG and NCG. Additionally, Pearson correlation coefficients were calculated to examine the relationships between anxiety, perceived stress, SBP and DBP within each group. A two-way repeated measures analysis of variance was conducted to determine whether the interventions significantly affected anxiety, SBP, and DBP. A chi-square test was used to assess whether the pass rates between the three groups were significantly different.

### 2.7 Ethical consideration

The studies involving human participants were reviewed and approved by the Medical Ethics Committee of The Air Force Hospital from Eastern Theater of the People’s Liberation Army (PLA). All participants provided written informed consent prior to participation.

## 3 Results

### 3.1 Descriptive statistics

Table 1 revealed no significant differences between the BBG and NCG in terms of anxiety and perceived stress (both *p* > 0.05). However, a substantial difference was observed between the groups regarding systolic blood pressure (SBP) and diastolic blood pressure (DBP), with the BBG group showing significantly higher values than the NCG group (*t* = 20.61, *p* < 0.001, *t* = 10.73, *p* < 0.001). This result demonstrates that the grouping is effective.

**TABLE 1:** Anxiety, perceived stress, SBP and DBP in BBG and NCG.

Characteristics	BBG (*n* = 102)	NCG (*n* = 108)	*t*	*df*	*p*
Anxiety	21.13 ± 4.61	20.65 ± 3.64	1.00	208	0.32
Perceived stress	30.69 ± 6.64	29.88 ± 6.00	0.93	208	0.36
SBP	146.65 ± 6.78	126.04 ± 8.04	20.12	208	<0.001
DBP	88.17 ± 6.89	77.59 ± 5.84	12.72	208	<0.001

### 3.2 Correlation analysis

Table 2 showed a significant correlation positive correlation between anxiety and perceived stress (*r* = 0.47, *p* < 0.001) within BBG; however, no significant correlation was found between SBP and DBP. Anxiety showed marginally significant correlation with both SBP and DBP (*r* = 0.13, *p* = 0.20; *r* = 0.15, *p* = 0.13). Additionally, perceived stress showed a significant correlation with both SBP and DBP (*r* = 0.20, *r* = 0.20, both *p* < 0.05). Similarly, within the NCG group detected a significant positive correlation between anxiety and perceived stress (*r* = 0.47, *p* < 0.001) and between SBP and DBP (*r* = 0.48, *p* < 0.001).

**TABLE 2:** Correlation between anxiety, perceived stress, SBP and DBP in BBG and NCG.

Group	Characteristics	Anxiety	Perceived stress	SBP	DBP
In BBG	Anxiety	1			
	Perceived stress	0.47 (0.000)***	1		
	SBP	0.13 (0.20)	0.20 (0.04)*	1	
	DBP	0.15 (0.14)	0.20 (0.04)*	0.02 (0.88)	1
In NCG	Anxiety	1			
	Perceived stress	0.47 (0.000)***	1		
	SBP	−0.02 (0.87)	0.03 (0.80)	1	
	DBP	−0.01 (0.96)	−0.10 (0.30)	0.48 (0.000)***	1

*P < 0.05,

***p < 0.001.

The further analysis of the blood pressure and anxiety changes in the high-pressure group revealed a significant negative correlation between the change in blood pressure and the change in anxiety.

### 3.3 Effect of intervention method and pre- and post-intervention on the anxiety, SBP and DBP

**FIGURE 2:**
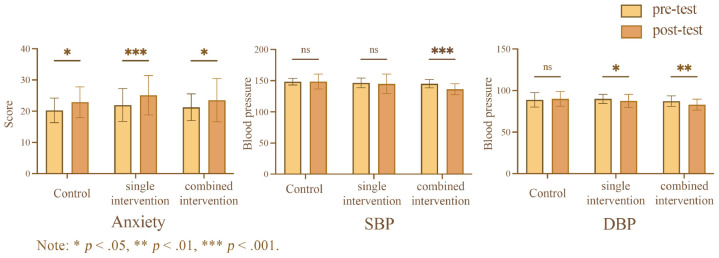
Effect of intervention method and pre- and post-intervention on the anxiety, SBP and DBP.

The results are presented in Figure 2 and Table 3, the results of two-way repeated measures ANOVA revealed a significant main effect of time on anxiety level was significant, anxiety levels in post-intervention (*M* = 23.84, SD = 0.61) were significantly higher than pre-intervention (*M* = 21.16, SD = 0.46), $F_{\left(1,99\right)}=23.37,p<0.001,\eta_{p}^{2}=0.19$. A Tukey-adjusted simple effects analysis was conducted to examine changes in anxiety levels from pre-test to post-test across the three groups. In the control group, scores significantly increased from 20.26 (pre-test) to 22.87 (post-test), with a least-squares (LS) mean difference of −2.61, 95% CI [4.60,0.63], t_(99)_ = 3.70, adjusted *p* < 0.05. The single intervention group also showed a significant increase, from 21.95 to 25.13, LS mean difference = −3.18, 95% CI [4.92,1.43], t_(99)_ 5.11, adjusted *p* < 0.001. Similarly, the combined intervention group exhibited a significant increase from 21.26 to 23.52, LS mean difference = −2.26, 95% CI [4.24,0.28], t_(99)_ = 3.20, adjusted *p* < 0.05.

**TABLE 3:** Effect of intervention method and pre- and post-intervention on the anxiety, SBP and DBP.

Characteristics	Intervention methods	Pre-intervention	Post-intervention	Repeated measures		
		M ± SD	M ± SD	Time [*F*(*p*)]	Group [*F*(*p*)]	Time Group [*F*(*p*)]
Anxiety	Control	20.26 ± 0.83	22.87 ± 1.10	23.37 (0.000)	1.62 (0.20)	0.25 (0.78)
	Single intervention	21.95 ± 0.73	25.13 ± 0.97			
	Combined intervention	21.26 ± 0.83	23.52 ± 1.10			
SBP	Control	148.39 ± 5.23	148.58 ± 1.10	7.08 (0.009)	7.65 (0.001)	4.37 (0.02)
	Single intervention	146.45 ± 7.75	144.85 ± 15.67			
	Combined intervention	145.16 ± 6.63	136.29 ± 8.75			
DBP	Control	88.77 ± 1.23	90.00 ± 1.42	5.56 (0.02)	4.17 (0.02)	4.03 (0.02)
	Single intervention	90.00 ± 1.08	87.53 ± 1.25			
	Combined intervention	87.23 ± 1.23	83.07 ± 1.42			

The results of two-way repeated measures ANOVA showed that the main effect of time on SBP was significant, SBP in post-intervention (*M* = 143.24, SD = 1.28) were significantly lower than pre-intervention (*M* = 146.67, SD = 0.67), $F_{\left(1,99\right)}=7.08,p<0.01,\eta_{p}^{2}=0.07$. A Tukey-adjusted simple effects analysis was conducted to examine changes in SBP from pre-test to post-test within each group. In the control group, there was no significant change in SBP (LS mean difference = −0.19, 95% CI [4.79, 4.41]), t_(99)_ = 0.12, adjusted *p* < 0.93. Similarly, the single intervention group showed no significant difference in SBP between pre-test and post-test (LS mean difference = 1.60, 95% CI [2.45, 5.65]), t_(99)_ = 1.11, adjusted *p* < 0.43. However, the combined intervention group demonstrated a significant reduction in SBP, with levels decreasing from 145.2 to 136.3 (LS mean difference = 8.87, 95% CI [4.27, 13.47]), t_(99)_ = 5.41, adjusted *p* < 0.001.

The results of two-way repeated measures ANOVA indicated a main effect of time on DBP was significant, DBP in post-intervention (*M* = 86.86, SD = 0.79) were significantly lower than pre-intervention (*M* = 88.67, SD = 0.68), $F_{\left(1,99\right)}=5.56,p<0.05,\eta_{p}^{2}=0.05$. A Tukey-adjusted simple effects analysis was conducted to examine changes in DBP from pre-test to post-test within each group. In the control group, there was no significant change in DBP (LS mean difference = −1.23, 95% CI [−3.96, 1.51]), t_99_ = 1.26, adjusted *p* = 0.38. The single intervention group showed a significant reduction in DBP between pre-test and post-test (LS mean difference = 2.48, 95% CI [0.07, 4.88]), t_99_ = 2.89, adjusted *p* < 0.05. Similarly, the combined intervention group demonstrated a significant decrease in DBP, with levels decreasing from 87.23 to 83.06 (LS mean difference = 4.16, 95% CI [1.43, 6.90]), t_99_ = 4.27, adjusted *p* < 0.01.

### 3.4 Effect of intervention methods on pass rates across three groups

As shown in Table 4, a chi-square test was conducted to assess the differences in the pass rates across three groups: the control group, the single-intervention group, and the combined-intervention group. The results, summarized in Table 4, revealed significant differences in the pass rates among the three groups, χ^2^(2) = 15.12, *p* = 0.001. Specifically, the pass rates in both the single-intervention group (57.5%) and combined-intervention group (74.2%) were significantly higher than those in the control group (25.8%). However, no significant difference was observed between the single-intervention group and the combined-intervention group.

**TABLE 4:** Pass counts and rates by intervention method.

Intervention methods	Pass (%)	Fail (%)	Total	χ^2^
Control	8 (25.8%)	23 (74.2%)	31	15.12**
Single intervention	23 (57.5%)	17 (42.5%)	40	
Combined intervention	23 (74.2%)	8 (25.8%)	31	

**P < 0.01.

## 4 Discussion

The present study uncovered the correlation between psychological factors and blood pressure, as well as the effects of different interventions on both variables. The results indicated that although anxiety and perceived stress levels were similar between the hypertension group and the normal blood pressure group, perceived stress was positively correlated with blood pressure in the hypertension group, while anxiety showed a marginally significant correlation with blood pressure. Intervention outcomes indicated that comprehensive psychological interventions effectively reduced both systolic and diastolic blood pressure, whereas single interventions yielded limited effects. Additionally, compliance rates improved across all intervention groups, with the comprehensive intervention demonstrating the most significant effectiveness.

### 4.1 Psychological factors and blood pressure correlations in BBG and NCG

The findings revealed that in the BBG group, perceived stress was significantly positively correlated with blood pressure, and anxiety showed a marginally significant positive association, whereas no such patterns were observed in the NCG group. This is partially consistent with the results of some previous studies (Forte et al., 2022; Gavrilova and Zawadzki, 2023). One possible explanation for this phenomenon is heightened hypothalamic-pituitary-adrenal (HPA) axis activation in individuals with hypertension, likely due to their prolonged near-hypertensive state, which increases their susceptibility to stress and anxiety (Johnson, 2019; Qi et al., 2023). Therefore, the BBG group may show a correlation between psychological factors and blood pressure, while the NCG group does not. Another possible explanation is that hypertension is often comorbid with trait or chronic anxiety (Rosas et al., 2021, 2024). Concurrently, individuals with higher levels of trait anxiety are more likely to show high state anxiety and stress (Weger and Sandi, 2018; Saviola et al., 2020). As the participants in the NCG group had normal blood pressure, their trait anxiety levels might generally be lower, resulting in weaker associations with state anxiety and perceived stress levels. This could help explain the absence of significant relationships between blood pressure and anxiety or stress levels within this group. Furthermore, additional potential psychological factors, such as emotional regulation ability and personality traits, or physiological mechanisms, including vascular elasticity and heart rate variability (HRV), may also act as influence factors between normotensive and prehypertensive groups (Piotrowska-Półrolnik et al., 2019; Chapuis-de-Andrade et al., 2021; Yugar et al., 2023; Kim, 2023). These differences warrant further investigation.

### 4.2 The impact of different intervention methods on blood pressure

Post-intervention results indicated that both relaxation training alone and its combination with cognitive intervention significantly reduced blood pressure compared to the control group, with the combined intervention demonstrating the most pronounced effect. Furthermore, intervention groups showed a significantly higher rate of achieving target blood pressure levels. These findings support previous research showing that psychological interventions can help manage blood pressure (Huang et al., 2023; Chen et al., 2024). Possible mechanisms underlying these effects can be explained by the BERN framework, an operational model within mind-body medicine (MBM) that encompasses four multimodal intervention strategies: Behavior, Exercise, Relaxation, and Nutrition (Esch and Stefano, 2022). Based on this framework, psychological interventions including relaxation training may reduce blood pressure through two main mechanisms. First, relaxation training modulates the autonomic nervous system by enhancing parasympathetic activity and reducing sympathetic arousal, thereby lowering heart rate, decreasing vascular resistance, and inducing a self-regulated relaxation response. Second, it activates the central nervous system’s rewards and motivation circuits, engaging self-regulation processes and neurobiological signaling pathways, including the release of key messengers such as nitric oxide (NO) and dopamine, which further reduce stress responses and promote cardiovascular relaxation. This study found that the combination of relaxation training and cognitive intervention yielded the most effective results in blood pressure management, providing empirical support for future interventions. Future research could further explore and compare the effectiveness of other interventions, such as forest bathing, mindfulness, and the combination of relaxation training and cognitive intervention, in their impact on blood pressure (Lee et al., 2020; Peterfalvi et al., 2021).

### 4.3 Anxiety’s role in psychological interventions for blood pressure reduction

It is noteworthy that participants’ anxiety levels increased after the intervention, which is contrary to the findings of previous research studies (Wells et al., 2021; White et al., 2022). There are several possible explanations for this phenomenon. Firstly, it is possible that the intervention may have altered participants’ cognitive and expressive perceptions of anxiety, leading to a more sensitive and accurate self-report of anxiety, rather than a true increase in anxiety (Van Der Gucht et al., 2019). Secondly, Participants’ awareness that failure to achieve the required blood pressure standard in a single test could result in disqualification from the selection process may have increased their stress levels, leading to increased anxiety. Despite elevated anxiety levels, participants exhibited a decrease in blood pressure, suggesting that psychological interventions may not rely solely on anxiety reduction to impact blood pressure (Mann, 2012). Indeed, some studies have suggested that relaxation training could directly regulate physiological responses, such as reducing sympathetic nervous system activity and improving cardiovascular function (Barnes et al., 2001; Rusinova et al., 2024). This would therefore influence blood pressure independently of psychological stress or anxiety relief (Ketelhut et al., 2023). It is worth noting that there is a small difference in the level of anxiety increase between different intervention methods, indicating that differences in intervention methods may affect changes in anxiety levels. Although the overall anxiety level increased, the anxiety level of subjects who received relaxation training combined with cognitive intervention increased less than that of subjects who only received relaxation training. Some previous studies have also suggested that relaxation training and interventions combined with cognitive measures are effective in reducing anxiety (Smits et al., 2008; Montero-Marin et al., 2019; Roper et al., 2024). Based on previous studies, this study concluded that compared with single relaxation training combined with cognitive psychological intervention, it is more effective in lowering blood pressure and relieving anxiety.

## 5 Limitation

There are several limitations in the study. First, the study lacks follow-up data, so it is unclear whether the effects of the intervention are short term or long term. Future research should include follow-up assessments. Second, the study only focused on the relationship between anxiety and blood pressure, but other psychological factors may also affect blood pressure. Future studies could look at other factors. Third, the study only used questionnaires and experimental methods. Using other methods could get a broader perspective in the future study. Fourth, the durations of the single and combined psychological interventions differed. Although there was a certain interval between the interventions and the second measurement, the difference in intervention duration could still potentially affect blood pressure readings.

## 6 Conclusion

In conclusion, the findings of this study, which identified a correlation between blood pressure and anxiety-stress perception in individuals with hypertension, provide compelling evidence in support of the efficacy of combined cognitive and relaxation interventions in the management of anxiety and regulation of blood pressure.

## Data Availability

The raw data supporting the conclusions of this article will be made available by the authors, without undue reservation.
